# *Plasmodium falciparum msp*1 and *msp*2 genetic diversity and allele frequencies in parasites isolated from symptomatic malaria patients in Bobo-Dioulasso, Burkina Faso

**DOI:** 10.1186/s13071-018-2895-4

**Published:** 2018-05-30

**Authors:** Anyirékun Fabrice Somé, Thomas Bazié, Issaka Zongo, R. Serge Yerbanga, Frédéric Nikiéma, Cathérine Neya, Liz Karen Taho, Jean-Bosco Ouédraogo

**Affiliations:** Institut de Recherche en Sciences de la Santé, Direction Régionale de l’Ouest, 399 Avenue de la Liberté, 01 BP 545, Bobo-Dioulasso, 01 Burkina Faso

**Keywords:** *Plasmodium falciparum*, *msp1*, *msp2*, Burkina Faso

## Abstract

**Background:**

In Burkina Faso, malaria remains the overall leading cause of morbidity and mortality accounting for 35.12% of consultations, 40.83% of hospitalizations and 37.5% of deaths. Genotyping of malaria parasite populations remains an important tool to determine the types and number of parasite clones in an infection. The present study aimed to evaluate the merozoite surface protein 1 (*msp1*) and merozoite surface protein 2 (*msp2*) genetic diversity and allele frequencies in Bobo-Dioulasso, Burkina Faso.

**Method:**

Dried blood spots (DBS) were collected at baseline from patients with uncomplicated malaria in urban health centers in Bobo-Dioulasso. Parasite DNA was extracted using chelex-100 and species were identified using nested PCR. *Plamodium falciparum msp1* and *msp2* genes were amplified by nested polymerase chain reaction (PCR) and PCR products were analyzed by electrophoresis on a 2.5% agarose gel. Alleles were categorized according to their molecular weight.

**Results:**

A total of 228 blood samples were analyzed out of which 227 (99.9%) were confirmed as *P. falciparum-*positive and one sample classified as mixed infection for *P. malaria* and *P. falciparum.* In *msp1*, the K1 allelic family was predominant with 77.4% (162/209) followed respectively by the MAD20 allelic family with 41.3% and R033 allelic family with 36%. In *msp2*, the 3D7 allelic family was the most frequently detected with 93.1 % compared to FC27 with 41.3%. Twenty-one different alleles were observed in *msp1* with 9 alleles for K1, 8 alleles for MAD20 and 4 alleles for R033. In *msp2*, 25 individual alleles were detected with 10 alleles for FC27 and 15 alleles for 3D7. The mean multiplicity of *falciparum* infection was 1.95 with respectively 1.8 (1.76–1.83) and 2.1 (2.03–2.16) for *msp1* and *msp2* (*P* = 0.01).

**Conclusions:**

Our study showed high genetic diversity and allelic frequencies of *msp1* and *msp2* in *Plasmodium falciparum* isolates from symptomatic malaria patients in Bobo-Dioulasso.

## Background

Despite substantial efforts to eliminate or control malaria, it remains the leading cause of morbidity and mortality in the world [1]. In Burkina Faso, malaria is responsible of 35.12% of consultations, 40.83% of hospitalizations and 37.5% of deaths. Since 2005, several efforts have been made in the country to reduce the burden of malaria including provision of artemisinin-based combinations treatments (ACTs), distribution of long-lasting insecticidal nets (LLINs) and scale-up of seasonal malaria chemoprevention with amodiaquine-sulfadoxine-pyrimethamine (AQ-SP) in children aged 6–59 months. However, in 2015, the Burkinabe health facilities recorded 7,836,411 malaria cases, including 450,042 cases of severe malaria and 5379 deaths [2].

Genotyping of malaria parasite populations remains an important tool to determine the types and number of parasite clones in an infection. In molecular epidemiological studies of malaria, this approach is used to investigate the genetic diversity of infections with consideration of various factors including transmission intensity and host immunity. The most widely used techniques for genotyping malaria infections are based on amplification by PCR of the polymorphic genes encoding the merozoite surface proteins 1 and 2 (MSP1 and MSP2) and the glutamate-rich protein (GLURP) [3–9]. MSP 1 and MSP2 are two major *Plasmodium falciparum* blood-stage malaria vaccine targets [10] playing important role in identification of genetically distinct *P. falciparum* parasite sub-populations [11]. They are involved in erythrocyte invasion [12] and are targeted by the immune responses [13, 14]. MSP1 is a 190 KDa surface protein encoded by the *msp1* gene located on chromosome 9 and contains 17 blocks of sequences flanked by conserved regions [15, 16]. Block 2, which is the most polymorphic part of MSP1, is grouped into three allelic families namely K1, MDA20 and R033 [17]. MSP2 is a glycoprotein encoded by the *msp2* gene located on chromosome 2 and composed by five blocks of which the central block is the most polymorphic [18]. The *msp2* alleles are grouped into two allelic families, FC27 and 3D7/IC1 [11].

Both *msp1* and *msp2* have been shown to be highly polymorphic in different geographical settings in malaria endemic countries [3, 7, 19, 20]. In areas with intense malaria transmission, the probability of new infection with the same parasite genotype in a subject is very low [21]. The diversity of the *P. falciparum* genome enables the comparison of the genotypes of isolates collected before the treatment and at the time of recurrent malaria infection. This comparison has been widely used to classify treatment outcomes in antimalarial clinical trials [22, 23]. To our knowledge, there is very limited information on *msp1* and *msp2* genetic diversity in Bobo-Dioulasso. The present study aimed to evaluate the genetic diversity and allele frequencies of *msp1* and *msp2* in malaria parasites isolated from symptomatic patients in Bobo-Dioulasso, Burkina Faso.

## Methods

### Study site

Samples for this study were provided by a clinical trial conducted from October to December 2012 in two health facilities of Colsama and Sakaby in the district of Do in Bobo-Dioulasso, located in the western region of Burkina Faso. Malaria transmission is holoendemic with a peak around August to October and an estimated entomological inoculation rate (EIR) of 300–500 infective bites per person per year. In 2012, the population of the health district of Do was estimated to be about 504,895 inhabitants [24].

### Patients and inclusion criteria

All subjects aged 6 months or more and attending Colsama and Sakaby health centers with fever or history of fever in the last 24 h were referred by a clinician for screening of malaria infection using Giemsa-stained thick and thin blood smears. Participants with *Plasmodium* spp. infection at parasite densities between 2000 and 200,000 parasites/μl and hemoglobin > 5g/dl were included in the clinical study after they or their parents/guardians had signed an informed consent form. Other inclusion criteria were: absence of known adverse events to study drugs, absence of non-malarial febrile diseases, absence of documented malaria treatment in the two weeks prior to enrolment, absence of danger signs or severe malaria. Details of the overall clinical study have been described elsewhere [25].

### Samples collection and laboratory analysis

For all patients included in the clinical study, blood was also collected on filter papers (Whatman 3 mm, GE Healthcare, Pittsburg, USA) at any scheduled or unscheduled visit, labeled, air-dried and stored into sealed plastic bags at ambient temperature. Parasite DNA was subsequently extracted from day 0 samples using the QIAamp DNA Mini Kit (Qiagen, Hilden, Germany) according to the Qiagen-DNA purification from dried blood spot protocol.

*Plasmodium* species were detected using a modified protocol from Snounou et al. [26]. Briefly, species were analyzed/determined by amplification of *18S* ribosomal RNA using nested PCR with secondary primers specific to the species *Plasmodium falciparum*, *Plasmodium malariae*, *Plasmodium ovale* and *Plasmodium vivax*. Primary and nested PCR primer sets used are listed in Table 1. Separate reactions were performed for each set of nested primers. For quality control, a template-free control was used in all reactions and genomic DNA from laboratory strains was used as a positive control for respective species. Both primary and secondary PCRs were carried out in a final volume of 25 μl containing 16.05 μl of sterile water, 0.5 μl of each primer (www.eurogentec.com), 2.5 μl of 10× PCR buffer, 2.5 μl of each dNTP, 1.5 μl of 25 mM MgCl_2_, 0.2 μl of Go Taq DNA polymerase (www.promega.com) and 2 μl of Template DNA or primary PCR product. All PCRs were performed in a BIO-RAD S1000 thermal cycler (Bio-Rad, California, USA) under the following conditions for primary (35 cycles) and secondary PCR (30 cycles): initial denaturation at 94 °C for 1 min, extension at 94 °C for 1 min, 58 °C for 2 min and 72 °C for 5 min, and final elongation at 72 °C for 5 min.

**Table 1:** Primary and secondary PCR primers for *Plasmodium* species

PCR round	Primer name	Primer sequence (5'-3')
Primary PCR	rPLUf	TTAAAATTGTTGCAGTTAAAACG
	rPLUr	CCTGTTGTTGCCTTAAACTTC
Nested PCR species	rFALf	TTAAACTGGTTTGGGAAAACCAAATATATT
	rFALr	ACACAATGAACTCAATCATGACTACCCGTC
	rMALf	ATAACATAGTTGTACGTTAAGAATAACCGC
	rMALr	AAAATTCCCATGCATAAAAAATTATACAAA
	rVIVf	CGCTTCTAGCTTAATCCACATAACTGATAC
	rVIVr	ACTTCCAAGCCGAAGCAAAGAAAGTCCTTA
	rOVAf	ATCTCTTTTGCTATTTTTTAGTATTGGAGA
	rOVAr	GGAAAAGGACACATTAATTGTATCCTAGTG

The *msp1* and *msp2* of all *Plasmodium falciparum* parasites were amplified using sequence-specific primers according to a modified protocol previously described [3]. In summary, the primary reaction used a set of primers corresponding to the conserved regions of block 2 for *msp1* and block 3 for *msp2*. The second reaction primer set targets specific allelic families of *msp1* (MAD20, K1 and R033) or *msp2* (IC/3D7 and FC27). Reactions for each set of primary and nested primers were performed separately. A template-free control was used in all reactions and genomic DNA from cloned laboratory strains was used as a positive control for respective alleles. Cycling conditions for both *msp1* and *msp2* as well as primer sequences are summarized in Table 2.

**Table 2:** Primers sequences, cycling conditions and annealing temperature for *msp*1 and *msp*2 PCR

Gene	PCR round	Primer name	Sequence (5'-3')	Cycling conditions	Allelic family (annealing temperature)	Positive control
*msp1*	Primary	M1-OF:	CTAGAAGCTTTAGAAGATGCAGTATTG	Initial denaturation: 95 °C for 5 min; PCR: 25 cycles of 94 °C for 1 min, 61 °C for 45 s, 72 °C for 1.5 min; final elongation:72 °C for 5 min	61 °C	
		M1-OR:	CTTAAATAGTATTCTAATTCAAGTGGATCA			
	Secondary	M1- KF:	AATGAAGAAGAAATTACTACAAAAGGTGC	Initial denaturation: 95 °C for 5 min; PCR: 30 cycles of 94 °C for 45 s, allele-specific °C for 30s, 72 °C for 1 min; final elongation: 72 °C for 5 min	K1(61 °C)	3D7
		M1-KR:	GCTTGCATCAGCTGGAGGGCTTGCACCAGA			
		M1-MF:	AAATGAAGGAACAAGTGGAACAGCTGTTAC		MAD20 (61 °C)	HB3
		M1-MR:	ATCTGAAGGATTTGTACGTCTTGAATTACC			
		M1-RF:	TAAAGGATGGAGCAAATACTCAAGTTGTTG		R033 (63 °C)	R033
		M1-RR:	CATCTGAAGGATTTGCAGCACCTGGAGATC			
*msp2*	Primary	MSP2-1:	ATGAAGGTAATTAAAACATTGTCTATTATA	Initial denaturation: 94 °C for 5 min; PCR: 40 cycles of 94 °C for 1.5 min, 55 °C for 45 s, 72 °C for 1.5 min; final elongation: 72 °C for 10 min	(55 °C)	
		MSP2-4:	ATATGGCAAAAGATAAAACAAGTGTTGCTG			
	Secondary	A1:	GCAGAAAGTAAGCCTTCTACTGGTGCT	Initial denaturation: 94 °C for 2 min; PCR: 30 cycles of 94 °C for 1.5 min, 55 °C for 45 s, 72 °C for 1.5 min; final elongation: 72 °C for 10 min	IC/3D7 (55 °C)	3D7
		3D7 A2:	GATTTGTTTCGGCATTATTATGA			
		FC27 B1:	GCAAATGAAGGTTCTAATACTAATAG		FC27 (55 °C)	HB3
		FC27 B2:	GCTTTGGGTCCTTCTTCAGTTGATTC			

All PCR products were analyzed by electrophoresis on a 2.5% agarose gel. DNA fragments were stained with ethidium bromide and visualized by UV trans-illumination. The sizes of the amplicons were detected using a 100 bp DNA ladder (Promega, Madison, USA). *Plasmodium* species were identified by comparison of DNA fragments from the samples with DNA fragments from known positive controls (www.beiresource.org). Alleles of *msp1* and *msp2* were categorized according to their molecular weights.

### Multiplicity of infection (MOI)

MOIs were calculated by dividing the total number of distinct *msp1* and *msp2* genotypes by the number of positive samples for each marker. The mean MOI was calculated by dividing the total number of alleles detected in both *msp1* and *msp2* by the total number of positive samples for both markers. Samples were considered single infected when harboring only one allele at each of the genotyped loci. Multiclonal infections were defined as infections with more than one allele in at least one locus.

### Statistical analysis

Data were collected with Epidata and analyzed by R version 3.4.0 (2017-04-21). The allelic frequency of *msp1* and *msp2* was calculated as the proportion of the allele detected for each allelic family out of the total of alleles detected. The frequency of polyclonal infection was calculated using number of samples with more than one amplified fragment out of the total samples. The mean MOI was determined by dividing the total number of alleles detected in both *msp1* and *msp2* by the total number of positive samples for both markers. The Chi-square test was used to compare proportions. Statistical significance was defined as *P* < 0.05.

## Results

### Baseline characteristics

Overall, 441 subjects were screened for malaria infection out of which 244 subjects were included in the clinical study. Sixteen subjects were excluded (withdraw of consent, protocol violation, loss of follow-up) and the remaining 228 subjects completed the study follow-up. Details on baseline characteristics of the study population will be published elsewhere. Briefly, the study included 110 male and 118 female subjects. The mean age was 9.8 years (± 0.6) with minimum and maximum ages of 0.6 and 60 years, respectively. The mean body axillary temperature measured prior to blood sampling was 38.51 °C (± 1.1) and the mean parasite count was 52,543 parasites (46,385.36–58,700.89) per μl of blood.

### Prevalence of *Plasmodium* species

All 228 blood samples analyzed were confirmed as *P. falciparum-*positive by nested-PCR. Only one sample was classified as a mixed infection for *P. malariae* and *P. falciparum*. *Plasmodium ovale* and *P. vivax* were not detected in the samples.

### Frequency of *msp1* and *msp2* allelic families

Out of 228 baseline samples analyzed, 209 were successfully amplified for *msp1* (91.67%) and 188 (82.8%) for *msp2*. In *msp1*, the K1 allelic family was predominant with 77.4% (162/209), followed by the MAD20 allelic family with 41.3% (86/209) and the R033 allelic family with 36% (75/209). Thirty-eight percent (38.1%) of the samples positive for *msp1* were classified as monoclonal infection and the remaining 61.9% were classified as polyclonal infections with K1/MAD20, K1/R033 and MAD20/R033 representing 21.5, 22.3 and 11.3%, respectively. Only 6.9% of samples harbored together the K1, MAD20 and R033 allelic families (Table 3). In *msp2*, the 3D7 allelic family was the most frequently detected with 88.2% (176/188) compared to FC27 with 52.9%. Allelic distributions of both *msp1* and *msp2* did not differ significantly when the study population was subdivided by age group (Table 4). About one-third (34.4%) of samples were classified as polyclonal infection and two-thirds classified as monoclonal infection for *msp2* (Table 5).

**Table 3:** Genetic diversity of *Plasmodium falciparum msp1* and *msp2*

Gene	Allele	Baseline sample			
		Positive *n*/*N* (%)	*P*-value	Fragment size (bp)	No. of alleles
*msp1*	MAD20	86/209 (41.14)	< 0.0001	160–300	8
	K1	162/209 (77.51)		160–350	9
	R033	76/209 (36.36)		130–220	4
	MAD20/K1	53/209 (21.45)			
	MAD20/R033	28/209 (11.33)			
	K1/R033	55/209 (22.26)			
	MAD20/K1/R033	17/209 (6.88)			
*msp2*	FC27	78/188 (41.26)	< 0.0001	250–530	10
	IC1/3D7	176/188 (93.12)		100–450	15
	FC27/IC1/3D7	65/188 (34.39)			

*Abbreviations*: *N* number of total samples analyzed, *n* number of positive samples

**Table 4:** Prevalence of *msp1* and *msp2* allelic families per age group

Age group	K1 *n* (%)	MAD *n* (%)	RO33 *n* (%)	3D7 *n* (%)	FC27 *n* (%)
< 5 years	55 (77.5)	29 (40.9)	25 (35.2)	59/62 (95.2)	28/62 (45.2)
> 5 years	106 (76.9)	57 (41.3)	51 (37.0)	116/126 (92.0)	50/126 (39.7)
*P-*value	0.9	0.9	0.7	0.3	0.3

**Table 5:** Multiplicity of infection of *msp1* and *msp2* in symptomatic malaria patients in Bobo-Dioulasso

Gene	MOI	Monoclonal infection % (*n*/*N*)	Polyclonal infection % (*n*/*N*)
*msp*1	1.8	38.1 (56/209)	61.9 (153/209)
*msp*2	2.1	65.6 (123/188)	34.4 (65/188)
*P*-value	0.01	< 0.0001	< 0.0001

*Abbreviations*: *N* number of samples successfully genotyped for *msp1* or *msp2*; *n* number of samples with monoclonal or multiclonal infections for *msp1* or *msp2*

### Genetic diversity and allelic frequency

**Fig. 1:**
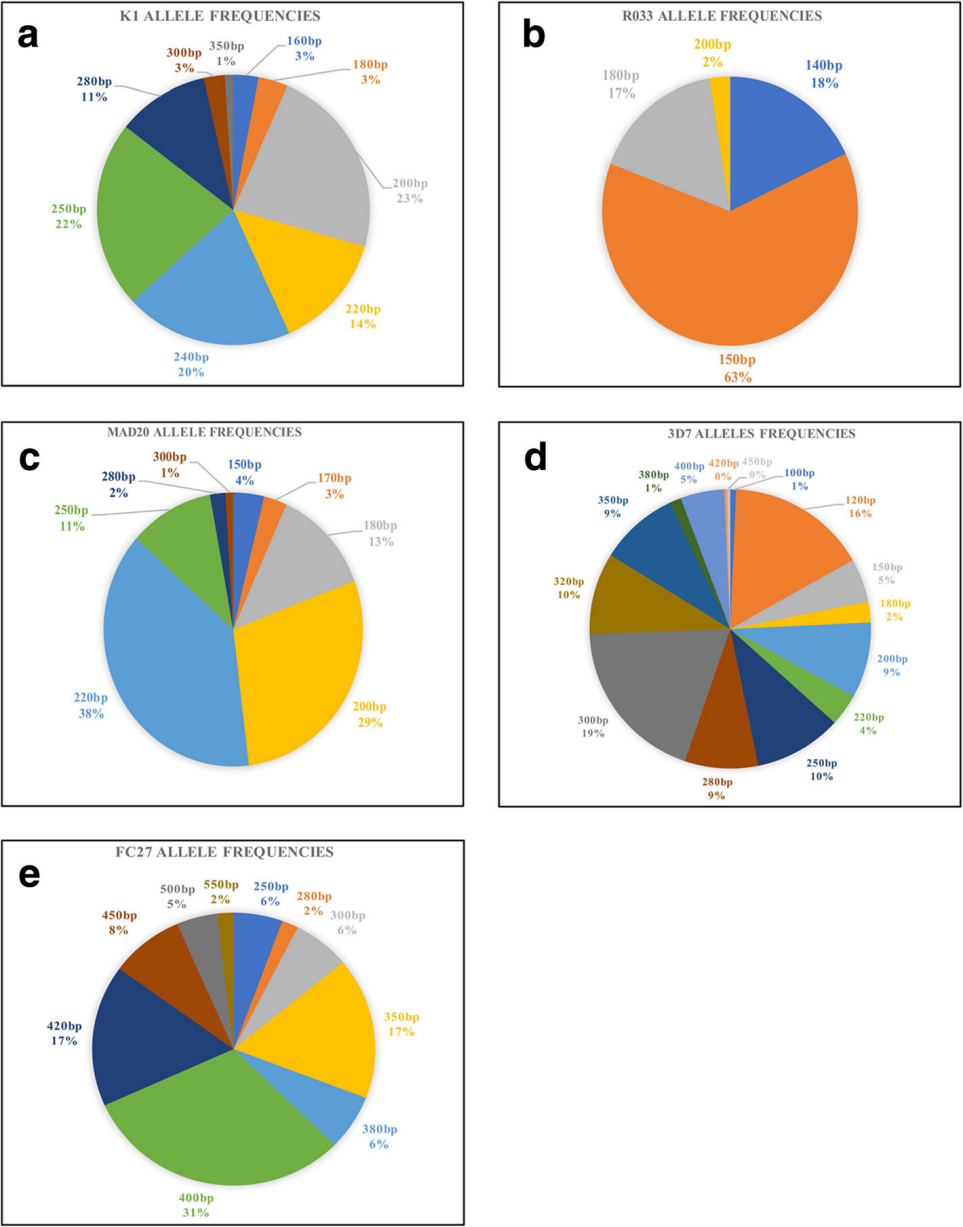
Allelic frequencies of *msp1* and *msp2* (**a**-**c** frequencies of *msp1* allelic families, **d**-**e** frequencies of *msp2* allelic families)

For both *msp1* and *msp2*, alleles were classified according to the size of the amplified PCR fragment. Twenty-one different alleles were observed in *msp1* with 9 alleles for K1 (fragment range 160–350 bp), 8 alleles for MAD20 (fragment range 150–300 bp) and for 4 alleles for R033 (fragment range 120–200 bp). In *msp2*, the K1 200 bp, 250 bp and 240 bp; the MAD20 200 bp and 220 bp; and the R033 150 bp were the most prevalent with a frequency above 20%. The allele distribution of *msp1* is represented in Fig. 1a-c.

With regard to *msp2*, 25 individual alleles were detected with 10 alleles for FC27 (fragment range 250–550 bp) and 15 alleles for 3D7 (fragment range 100–450 bp). The frequency of all alleles detected in the 3D7 family was less than 20% while the allele with 400 bp (31%) was the most prevalent in the FC27 family. Allele distribution for both FC27 and 3D7 families is illustrated in Fig. 1d-e.

### Multiplicity of infection (MOI)

In total, 61.9% (153/209) and 34.4% (65/188) of the isolates contained multiple infections in *msp1* and *msp2*, respectively. The MOIs for both *msp1* and *msp2* are summarized in Table 5. MOI for *msp2* was 2.1 (2.03–2.16) with a range of 1–6 strains, and was higher than MOI for *msp1*, i.e. 1.8 (1.76–1.83) with a range of 1–4 strains (*P* = 0.01).

## Discussion

*Plasmodium falciparum msp1* and *msp2* are currently being recommended in antimalarial clinical trials as standard markers to distinguish recrudescent from newly infecting malaria parasites [27]. However, to our knowledge, very few studies have investigated the genetic diversity of *msp1* and *msp2* in malaria parasites circulating in many endemic countries including Burkina Faso. Our study aimed to evaluate the genetic diversity and allelic frequency of *msp1* and *msp2* in malaria parasites isolated from symptomatic patients in Bobo-Dioulasso.

Our study showed that the frequency of successfully genotyped samples for *msp1* was higher than *msp2*. This result is consistent with previous studies in a rural area of Burkina Faso [28] and in Cote d’Ivoire and Gabon [29]. K1 and 3D7 were the predominant families for *msp1* and *msp2* as also demonstrated in previous studies in Africa including Burkina Faso [28, 30], Cote d’Ivoire, Gabon [29] and Ethiopia [11], but in contrast to other studies in Sudan and Central Africa [31]. These two allelic families might play important roles in clinical malaria at least in Bobo-Dioulasso. In our study, we did not examine the association between the dominant allelic families and the manifestation of the disease because all samples were collected from uncomplicated malaria patients. In previous studies, the dominance of K1 allelic family has been associated with severe malaria [32, 33] and also with asymptomatic malaria [34, 35].

Our study reported high genetic diversity with 21 and 25 genotypes for *msp1* and *msp2*, respectively, in parasites circulating in the southwest of Burkina Faso. This finding is consistent with those from previous studies in different malaria endemic countries in Africa [28, 36, 37]. The high parasite genetic diversity may be related to the intensity of malaria transmission in Bobo-Dioulasso where 767,126 cases were recorded in 2015 [2]. A number of studies have demonstrated that the majority of subsequent malaria episodes in this area are due to newly infecting parasites [38–40]. This high genetic diversity found in Bobo-Dioulasso might be associated with the high risk of being infected by multiple parasites haboring different genotypes. The genetic diversity reported could also be an effect of factors such as antimalarial pressure, the use of LLINs, insecticide indoor residual spraying and other factors as demonstrated in previous studies [28].

Our study did not show any significant difference in MOI using *msp1* and *msp2* with nearly two alleles per locus for both markers. The overall MOI reported in this study is lower than that reported by Soulama et al. [28] in parasites isolated from symptomatic children in Ouagadougou and Saponé, respectively located in the centre and centre-south of Burkina Faso [28]. The difference in MOI can be explained by the differences in geographical areas, intensity of malaria transmission and other factors such as the difference in age of study population and mean parasite density in the study population [31, 36, 41]. In our study, we did not analyze MOI according to the age, intensity of transmission or parasite density but several studies reported conflicting results. Some studies have demonstrated that MOI correlates with ages, parasite density [41, 42] and intensity of malaria transmission [43] but others studies failed to demonstrate this correlation [44, 45]. The low MOI reported in our study can also be explained as an effect of malaria control measure such as the deployment of artemisinin-combination therapy [46], and the large-scale distribution of LLINs across the country.

One limitation of this study is that the allelic frequencies and genetic diversity reported may have been estimated incorrectly due to the detection limit of the PCR technique used in the study. Alleles with short differences in length (less than 10 bp) might not be clearly distinguished as separate alleles on agarose gel and this could lead to a misclassification of the genotype.

## Conclusions

Our study showed high genetic diversity and allelic frequencies of *msp1* and *msp2* in *Plasmodium falciparum* isolates from uncomplicated malaria patients in Bobo-Dioulasso. K1 and 3D7 are the most prevalent allelic families. More than 50% of infections are classified as polyclonal infection for *msp1* against one-third percent for *msp2*. The high diversity reported may reinforce the use of *msp1* and *msp2* to distinguish recrudescent from new infection in antimalarial efficacy trials in Burkina Faso. However, further investigations with more powerful techniques such as capillary electrophoresis and DNA sequencing are needed to better characterize the malaria parasites in the country.

## Abbreviations

ACT: Artemisinin-based combinations therapy
AQ-SP: Amodiaquine-sulfadoxine-pyrimethamine
ARN: Acid ribonucleic
DBS: Dried blood spots
DNA: Deoxyribonucleic acid
EIR: Entomological inoculation rate
g/dl: Gram/deciliter
GLURP: Glutamate-rich protein
KDa: Kilodalton
LLIN: Long-lasting insecticidal nets
MOI: Multiplicity of infection
*msp1*: Merozoit surface protein
PCR: Polymerase chain reaction
UV: Ultraviolet

## References

[1] World Health Organization (2016). World malaria report 2016.

[2] Burkina Faso: Ministère de la Santé. Annuaire Statistique 2016. SG/DGESS. 2016. p. 342.

[3] Snounou G, Zhu X, Siripoon N, Jarra W, Thaithong S, Brown KN (1999). Biased distribution of msp1 and msp2 allelic variants in *Plasmodium falciparum* populations in Thailand. Trans R Soc Trop Med Hyg.

[4] Babiker H, Ranford-Cartwright L, Sultan A, Satti G, Walliker D (1994). Genetic evidence that RI chloroquine resistance of *Plasmodium falciparum* is caused by recrudescence of resistant parasites. Trans R Soc Trop Med Hyg.

[5] A-Elbasit IE, ElGhazali G, A-Elgadir TM, Hamad AA, Babiker HA, Elbashir MI, Giha HA (2007). Allelic polymorphism of MSP2 gene in severe *P. falciparum* malaria in an area of low and seasonal transmission. Parasitol Res.

[6] Felger I, Irion A, Steiger S, Beck HP (1999). Genotypes of merozoite surface protein 2 of *Plasmodium falciparum* in Tanzania. Trans R Soc Trop Med Hyg.

[7] Babiker HA, Lines J, Hill WG, Walliker D (1997). Population structure of *Plasmodium falciparum* in villages with different malaria endemicity in east Africa. Am J Trop Med Hyg.

[8] Babiker HA, Ranford-Cartwright LC, Currie D, Charlwood JD, Billingsley P, Teuscher T (1994). Random mating in a natural population of the malaria parasite *Plasmodium falciparum*. Parasitology.

[9] Bakhiet AM, Abdel-Muhsin AM, Elzaki SE, Al-Hashami Z, Albarwani HS, AlQamashoui BA (2015). *Plasmodium falciparum* population structure in Sudan post artemisinin-based combination therapy. Acta Trop.

[10] Chitarra V, Holm I, Bentley GA, Petres S, Longacre S (1999). The crystal structure of C-terminal merozoite surface protein 1 at 1.8 A resolution, a highly protective malaria vaccine candidate. Mol Cell.

[11] Mohammed H, Mindaye T, Belayneh M, Kassa M, Assefa A, Tadesse M (2015). Genetic diversity of *Plasmodium falciparum* isolates based on MSP-1 and MSP-2 genes from Kolla-Shele area, Arbaminch Zuria District, southwest Ethiopia. Malar J.

[12] Holder AA, Blackman MJ, Burghaus PA, Chappel JA, Ling IT, McCallum-Deighton N (1992). A malaria merozoite surface protein (MSP1)-structure, processing and function. Mem Inst Oswaldo Cruz.

[13] Apio B, Nalunkuma A, Okello D, Riley E, Egwang TG (2000). Human IgG subclass antibodies to the 19 kilodalton carboxy terminal fragment of *Plasmodium falciparum* merozoite surface protein 1 (MSP1(19)) and predominance of the MAD20 allelic type of MSP1 in Uganda. East Afr Med J.

[14] Woehlbier U, Epp C, Kauth CW, Lutz R, Long CA, Coulibaly B (2006). Analysis of antibodies directed against merozoite surface protein 1 of the human malaria parasite *Plasmodium falciparum*. Infect Immun.

[15] Takala S, Branch O, Escalante AA, Kariuki S, Wootton J, Lal AA (2002). Evidence for intragenic recombination in *Plasmodium falciparum*: identification of a novel allele family in block 2 of merozoite surface protein-1: Asembo Bay Area Cohort Project XIV. Mol Biochem Parasitol.

[16] Tanabe K, Mackay M, Goman M, Scaife JG (1987). Allelic dimorphism in a surface antigen gene of the malaria parasite *Plasmodium falciparum*. J Mol Biol.

[17] Farrar CT, DePeralta DK, Day H, Rietz TA, Wei L, Lauwers GY (2015). 3D molecular MR imaging of liver fibrosis and response to rapamycin therapy in a bile duct ligation rat model. J Hepatol.

[18] Smythe JA, Coppel RL, Day KP, Martin RK, Oduola AM, Kemp DJ (1991). Structural diversity in the *Plasmodium falciparum* merozoite surface antigen 2. Proc Natl Acad Sci USA.

[19] Silue KD, Felger I, Utzinger J, Beck HP, Smith TA, Tanner M (2006). Prevalence, genetic diversity and multiplicity of *Plasmodium falciparum* infection in school children in central Cote d'Ivoire. Med Trop (Mars).

[20] Falk N, Maire N, Sama W, Owusu-Agyei S, Smith T, Beck HP (2006). Comparison of PCR-RFLP and Genescan-based genotyping for analyzing infection dynamics of *Plasmodium falciparum*. Am J Trop Med Hyg.

[21] Snounou G, Beck HP (1998). The use of PCR genotyping in the assessment of recrudescence or reinfection after antimalarial drug treatment. Parasitol Today.

[22] Cattamanchi A, Kyabayinze D, Hubbard A, Rosenthal PJ, Dorsey G (2003). Distinguishing recrudescence from reinfection in a longitudinal antimalarial drug efficacy study: comparison of results based on genotyping of msp-1, msp-2, and glurp. Am J Trop Med Hyg.

[23] Zongo I, Dorsey G, Rouamba N, Dokomajilar C, Lankoande M, Ouedraogo JB (2005). Amodiaquine, sulfadoxine-pyrimethamine, and combination therapy for uncomplicated falciparum malaria: a randomized controlled trial from Burkina Faso. Am J Trop Med Hyg.

[24] de la Santé M (2013). Plan d’action 2013 du District Sanitaire de Do.

[25] Some AF, Sorgho H, Zongo I, Bazie T, Nikiema F, Sawadogo A (2016). Polymorphisms in K13, pfcrt, pfmdr1, pfdhfr, and pfdhps in parasites isolated from symptomatic malaria patients in Burkina Faso. Parasite.

[26] Snounou G, Viriyakosol S, Jarra W, Thaithong S, Brown KN (1993). Identification of the four human malaria parasite species in field samples by the polymerase chain reaction and detection of a high prevalence of mixed infections. Mol Biochem Parasitol.

[27] World Health Orgaization. Methods and techniques for clinical trials on antimalarial drug efficacy: genotyping to identify parasite population. Geneva: World Health Orgaization; 2008.

[28] Soulama I, Nebie I, Ouedraogo A, Gansane A, Diarra A, Tiono AB (2009). *Plasmodium falciparum* genotypes diversity in symptomatic malaria of children living in an urban and a rural setting in Burkina Faso. Malar J.

[29] Yavo W, Konate A, Mawili-Mboumba DP, Kassi FK, Tshibola Mbuyi ML, Angora EK (2016). Genetic polymorphism of msp1 and msp2 in *Plasmodium falciparum* isolates from Cote d'Ivoire *versus* Gabon. J Parasitol Res.

[30] Mwingira F, Nkwengulila G, Schoepflin S, Sumari D, Beck HP, Snounou G (2011). *Plasmodium falciparum* msp1, msp2 and glurp allele frequency and diversity in sub-Saharan Africa. Malar J.

[31] Hamid MM, Mohammed SB, El Hassan IM (2013). Genetic diversity of *Plasmodium falciparum* field isolates in Central Sudan inferred by PCR genotyping of merozoite surface protein 1 and 2. N Am J Med Sci.

[32] Ntoumi F, Mercereau-Puijalon O, Luty A, Georges A, Millet P (1996). High prevalence of the third form of merozoite surface protein-1 in *Plasmodium falciparum* in asymptomatic children in Gabon. Trans R Soc Trop Med Hyg.

[33] Kun JF, Schmidt-Ott RJ, Lehman LG, Lell B, Luckner D, Greve B (1998). Merozoite surface antigen 1 and 2 genotypes and rosetting of *Plasmodium falciparum* in severe and mild malaria in Lambarene, Gabon. Trans R Soc Trop Med Hyg.

[34] Amodu OK, Adeyemo AA, Ayoola OO, Gbadegesin RA, Orimadegun AE, Akinsola AK (2005). Genetic diversity of the msp-1 locus and symptomatic malaria in south-west Nigeria. Acta Trop.

[35] Babiker HA (1998). Unstable malaria in Sudan: the influence of the dry season. *Plasmodium falciparum* population in the unstable malaria area of eastern Sudan is stable and genetically complex. Trans R Soc Trop Med Hyg.

[36] Konate L, Zwetyenga J, Rogier C, Bischoff E, Fontenille D, Tall A (1999). Variation of *Plasmodium falciparum* msp1 block 2 and msp2 allele prevalence and of infection complexity in two neighbouring Senegalese villages with different transmission conditions. Trans R Soc Trop Med Hyg.

[37] Amodu OK, Oyedeji SI, Ntoumi F, Orimadegun AE, Gbadegesin RA, Olumese PE (2008). Complexity of the msp2 locus and the severity of childhood malaria, in south-western Nigeria. Ann Trop Med Parasitol.

[38] Zongo I, Dorsey G, Rouamba N, Dokomajilar C, Sere Y, Rosenthal PJ (2007). Randomized comparison of amodiaquine plus sulfadoxine-pyrimethamine, artemether-lumefantrine, and dihydroartemisinin-piperaquine for the treatment of uncomplicated *Plasmodium falciparum* malaria in Burkina Faso. Clin Infect Dis.

[39] Zongo I, Dorsey G, Rouamba N, Tinto H, Dokomajilar C, Guiguemde RT (2007). Artemether-lumefantrine versus amodiaquine plus sulfadoxine-pyrimethamine for uncomplicated falciparum malaria in Burkina Faso: a randomised non-inferiority trial. Lancet.

[40] Some AF, Sere YY, Dokomajilar C, Zongo I, Rouamba N, Greenhouse B (2010). Selection of known *Plasmodium falciparum* resistance-mediating polymorphisms by artemether-lumefantrine and amodiaquine-sulfadoxine-pyrimethamine but not dihydroartemisinin-piperaquine in Burkina Faso. Antimicrob Agents Chemother.

[41] Vafa M, Troye-Blomberg M, Anchang J, Garcia A, Migot-Nabias F (2008). Multiplicity of *Plasmodium falciparum* infection in asymptomatic children in Senegal: relation to transmission, age and erythrocyte variants. Malar J.

[42] Slater M, Kiggundu M, Dokomajilar C, Kamya MR, Bakyaita N, Talisuna A (2005). Distinguishing recrudescences from new infections in antimalarial clinical trials: major impact of interpretation of genotyping results on estimates of drug efficacy. Am J Trop Med Hyg.

[43] Bendixen M, Msangeni HA, Pedersen BV, Shayo D, Bodker R (2001). Diversity of *Plasmodium falciparum* populations and complexity of infections in relation to transmission intensity and host age: a study from the Usambara Mountains, Tanzania. Trans R Soc Trop Med Hyg.

[44] Basco LK, Ringwald P (2001). Molecular epidemiology of malaria in Yaounde, Cameroon. VIII. Multiple *Plasmodium falciparum* infections in symptomatic patients. Am J Trop Med Hyg.

[45] Issifou S, Ndjikou S, Sanni A, Lekoulou F, Ntoumi F. No influence of the transmission season on genetic diversity and complexity of infections in *Plasmodium falciparum* isolates from Benin. Afr J Med Med Sci. 2001;30(Suppl.):17–20. 14513933

[46] Gansane A, Nebie I, Soulama I, Tiono A, Diarra A, Konate AT (2009). Change of antimalarial first-line treatment in Burkina Faso in 2005. Bull Soc Pathol Exot.

